# Compositional Variability of Rowanberry Genotypes and Sugar–Organic Acid Distribution Between Juice and Pomace

**DOI:** 10.3390/molecules31162796

**Published:** 2026-08-11

**Authors:** Viive Sarv, Hedi Kaldmäe, Alar Aluvee

**Affiliations:** Polli Horticultural Research Centre, Institute of Agricultural and Environmental Sciences, Estonian University of Life Sciences, Uus 2, Polli, Mulgi Parish, 69108 Viljandi, Estonia; hedi.kaldmae@emu.ee (H.K.); alar.aluvee@emu.ee (A.A.)

**Keywords:** *Sorbus* spp., rowanberry, sorbitol, organic acids, pomace, cultivar variability, valorisation

## Abstract

This study evaluated the compositional and processing characteristics of 17 rowanberry (*Sorbus* spp.) genotypes, including sweet cultivars, interspecific hybrids, and a wild rowanberry. Fruit weight, colour parameters, soluble solids content (SSC), and titratable acidity (TA) were determined to assess fruit quality, and the distribution of soluble sugars and organic acids among whole fruit, juice, and pomace fractions was investigated. Significant (*p* < 0.05) variation was observed among genotypes, with fruit weight ranging from 0.40 g in wild rowanberry to 1.55 g in ‘Alaja Krupnaja’. Hierarchical cluster analysis grouped the genotypes into two major clusters according to colour characteristics. Based on the SSC/TA ratio, ‘Burka’ and ‘Oranzevaja’ exhibited the most favourable balance between sweetness and acidity. Sorbitol was the predominant soluble carbohydrate and showed pronounced cultivar-dependent variation, ranging from 46.2 mg/g DW in ‘Likernaja’ to 189.0 mg/g DW in ‘Krasnaja’. Malic acid was the dominant organic acid in all fractions, reaching 70.9 mg/g DW in the pomace of ‘Krasnaja’. This study provides the first comprehensive comparison of sugar and organic acid partitioning among fruit, juice, and pomace fractions in a diverse set of rowanberry genotypes, revealing cultivar-specific patterns of carbon allocation relevant to fruit quality, processing, and by-product valorisation.

## 1. Introduction

Rowanberry (*Sorbus* spp.) is an underutilised fruit species that has attracted increasing interest due to its adaptability to northern climates and its potential as a raw material for food processing [1,2]. In traditional medicine, fresh rowanberries, as well as syrups, teas, and alcoholic tinctures, have been used as appetite-stimulating, antidiarrheal, anti-inflammatory, and diuretic remedies [3,4]. Despite their high acidity and astringency, rowanberries possess considerable nutritional and functional potential and can be successfully used for juice production and other processed products, such as purées, jams, jellies, and marmalades [5,6].

Recently, growing consumer demand for clean-label foods has increased interest in natural colourants and antioxidant-rich fruit ingredients. Owing to their high content of phenolic compounds and naturally occurring pigments, rowanberries have attracted attention as a potential ingredient for jams and other fruit products [7]. According to Wojdyło et al. (2013), mixed jams prepared from rowanberries and other coloured berries can be a good source of antioxidants and can enhance pigment retention and colour stability in gelled jams [8].

In addition to colour and antioxidant properties, the technological and sensory quality of rowanberry products is influenced by the composition of sugars and organic acids [9,10,11]. These compounds contribute substantially to sweetness, acidity, and overall flavour perception, thereby affecting consumer acceptance [12]. Because the fruits of wild *S. aucuparia* are often characterised by a pronounced astringent and sour taste, breeding efforts have focused on improving fruit quality. Consequently, the Russian plant breeder Ivan Michurin and his co-workers developed interspecific hybrids of *S. aucuparia* with species of *Pyrus*, *Malus*, *Aronia*, and *Crataegus* to improve fruit quality [13].

Glucose and fructose are generally reported as the predominant sugars in most berry fruits [14,15]. However, Mikulic-Petkovsek et al. (2012) reported that rowanberry fruits exhibited the highest total sugar content among 25 analysed wild and cultivated berry species [12]. Owing to their relatively high sugar concentrations, some *Sorbus* genotypes may represent valuable ingredients for reducing the need for added sugars in fruit products. Indeed, total sugar contents reported for certain rowanberry cultivars exceed those of commonly processed fruits such as plum, peach, and apricot [16].

Most plant species primarily produce sucrose and starch during photosynthesis, with sucrose serving as the main transport carbohydrate. In contrast, many horticultural plants synthesise polyols in addition to sucrose and starch, and use both compounds for carbon translocation. In Rosaceae species, including rowanberries, sorbitol is the predominant polyol and serves as a major translocated photosynthate [17]. Studies on Rosaceae fruit trees have shown that sorbitol accumulation is associated with responses to abiotic stresses, including drought, salinity, low temperatures, and micronutrient deficiencies [18]. This may contribute to the adaptation of wild rowan trees to harsh Nordic climates. Sorbitol plays a central role in carbon transport and storage metabolism in species such as rowanberry, apple, and pear [19,20]. In addition to its physiological importance in plants, sorbitol is widely used as a sugar substitute because of its lower energy value, low glycaemic index, and reduced cariogenic potential compared with conventional sugars [21]. Despite the physiological and technological importance of sorbitol, comparative information on its distribution among rowanberry cultivars and processing fractions remains limited.

Soluble solids content and titratable acidity are widely used indicators of fruit maturity [22] and flavour balance [23]. Detailed sugar and organic acid profiles provide insight into metabolism and technological properties [24]. Together with sensory quality, the content and ratio of sugars and organic acids play a key role in determining fruit processing suitability [25]. In addition to the composition of fresh rowanberries, the distribution of compounds between juice and pomace fractions is important for evaluating processing efficiency and by-product utilisation.

In previous studies, the proximate composition, mineral and pectin content, fatty acid profile, phenolic composition, antioxidant capacity, and breeding background of sweet rowanberry cultivars were characterised [26,27]. However, information on the composition and distribution of soluble sugars, sorbitol, and organic acids across diverse rowanberry genotypes, as well as their partitioning between juice and pomace fractions, remains limited. Such information is important for assessing fruit quality, processing suitability, and the valorisation potential of rowanberry by-products. To our knowledge, no comparative study has systematically evaluated these characteristics in the whole fruits, juice, and pomace fractions of a wide range of rowanberry genotypes.

Therefore, the aim of this study was to evaluate the variability in the physical and chemical characteristics of 17 rowanberry genotypes, including sweet cultivars, interspecific hybrids, and a wild genotype (*S. aucuparia*). Particular emphasis was placed on fruit quality attributes, soluble sugar and organic-acid composition, and the distribution of these compounds among whole-fruit, juice, and pomace fractions in order to identify cultivars with favourable characteristics for food-processing applications and by-product valorisation.

## 2. Results

### 2.1. Variation in Fruit Characteristics

#### 2.1.1. Fruit Masses

**Figure 1:**
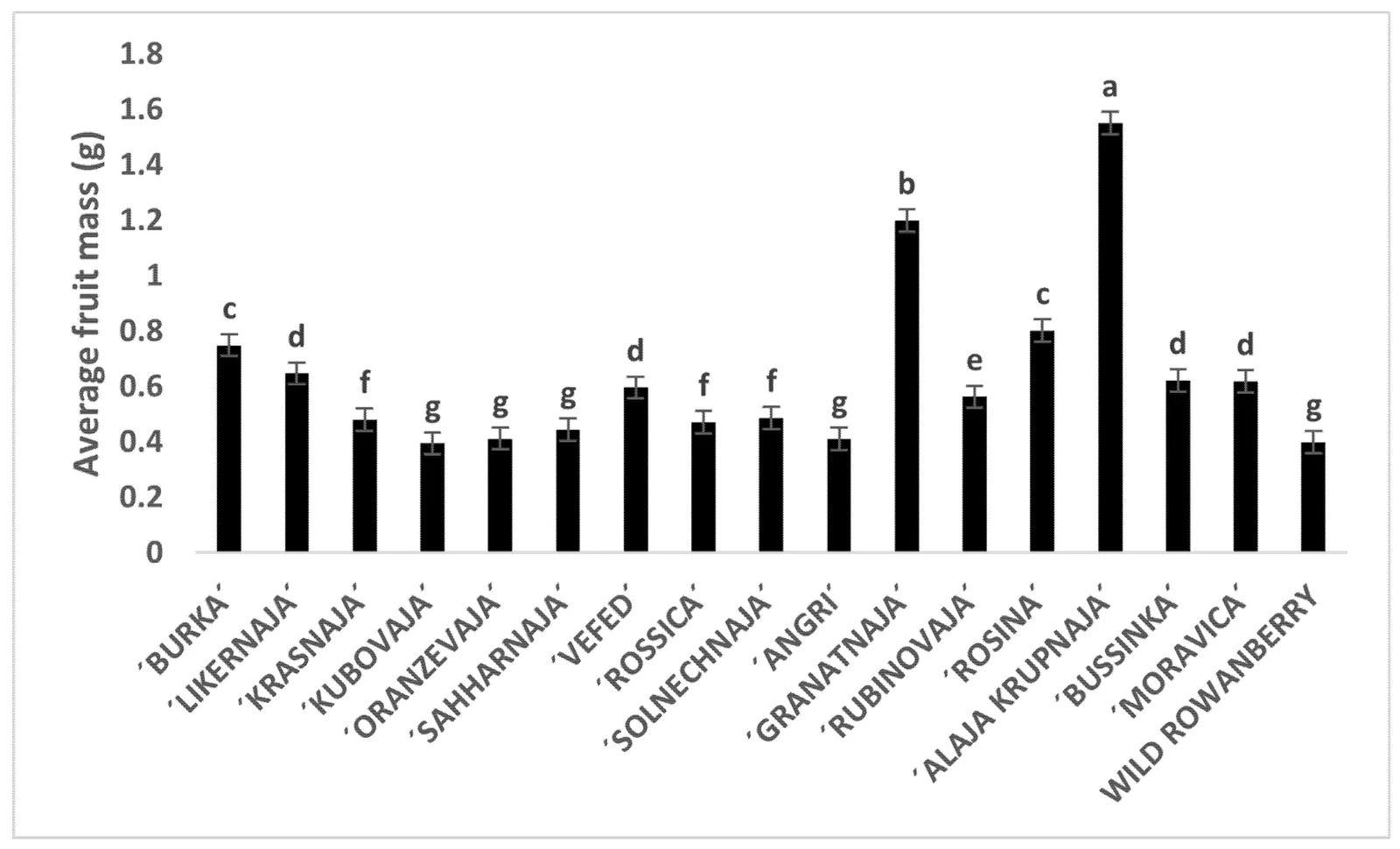
Average fruit mass of 17 rowanberry genotypes. Different letters indicate significant differences according to Tukey’s HSD test.

Fruit mass showed substantial variation among the rowanberry genotypes, ranging from 0.40 g in wild rowanberry (Sorbus aucuparia) to 1.55 g in ‘Alaja Krupnaja’ (Figure 1). The cultivars ‘Alaja Krupnaja’ and ‘Granatnaja’ produced significantly larger fruits than all other genotypes. A second group, comprising ‘Rosina’ and ‘Burka’, exhibited relatively high fruit mass. Most cultivars formed an intermediate group with overlapping values, indicating only limited statistical differentiation despite numerical variation.

The smallest fruits were recorded for ‘Sahharnaja’, ‘Oranzevaja’, ‘Angri’, and ‘Kubovaja’. Their fruit masses did not differ significantly from those of the wild rowanberry.

These results demonstrate that fruit size is strongly genotype dependent. They also highlight the potential of cultivar selection for increasing fruit mass while identifying cultivars with fruit sizes comparable to the wild type.

#### 2.1.2. Colour Parameters

**Figure 2:**
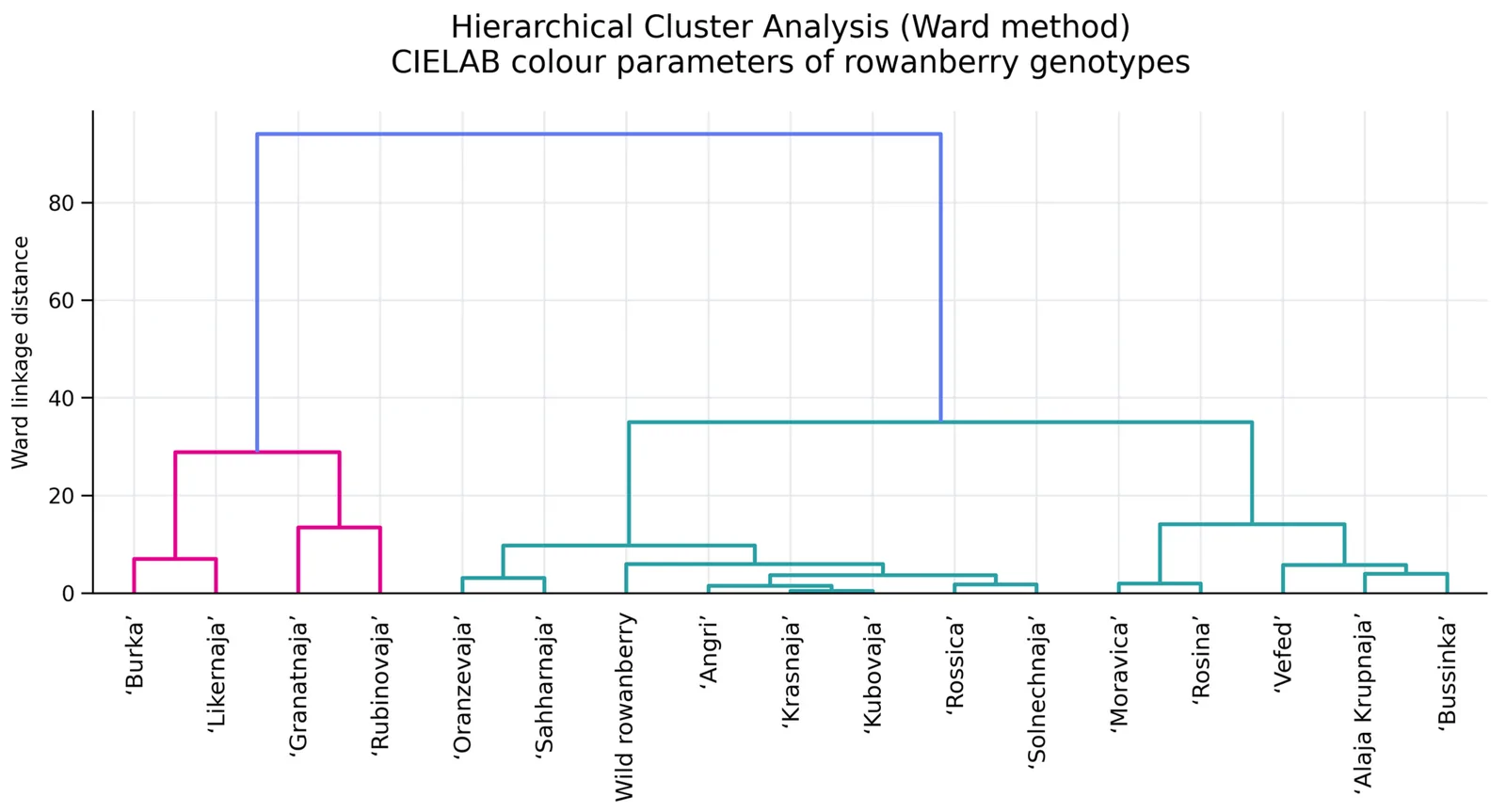
Hierarchical cluster analysis (Ward’s method) of 17 rowanberry genotypes based on CIELAB (*Commission Internationale de l’Éclairage Lab*). The dendrogram illustrates the grouping of genotypes according to fruit colour characteristics. Different branch colours are used solely to visually distinguish clusters.

During fruit maturation, colour changes markedly, and ripe fruits develop their characteristic cultivar-specific appearance. As one of the most important visual quality attributes, colour strongly influences food attractiveness, consumer expectations, and purchasing decisions [28]. Fruit color was evaluated using the CIELAB (Commission Internationale de l’Éclairage Lab) color space*, where L* represents lightness, a* the red-green axis, and b* the yellow-blue axis. Hierarchical cluster analysis based on CIELAB colour parameters separated the rowanberry genotypes into distinct groups according to fruit pigmentation characteristics (Figure 2). The dark-red cultivars ‘Burka’ and ‘Likernaja’, both hybrids between rowanberry and chokeberry, formed a clearly distinct cluster. Similar observations have been reported previously [29], where ‘Burka’ exhibited particularly low L* and C* values, indicating a dark and less saturated appearance. The red-fruited hybrid cultivars ‘Granatnaja’ and ‘Rubinovaja’ formed an intermediate cluster between the dark-red hybrids and the lighter orange-coloured cultivars. Most cultivars, including the wild rowanberry, grouped within a larger cluster characterised by higher lightness (L*) values and more orange hues. Cultivars such as ‘Alaja Krupnaja’, ‘Vefed’, ‘Moravica’, ‘Rosina’, and ‘Bussinka’, which exhibited high chroma values and intense red–orange pigmentation, occupied an intermediate position, reflecting more pronounced colour development than the wild genotype.

#### 2.1.3. Soluble Solids Content, Titratable Acidity, and Their Ratio

Soluble solids content and titratable acidity showed considerable variation among cultivars (Table 1). The balance between sugars and organic acids is a key determinant of fruit flavour and processing suitability. Soluble solids content (°Brix) reflects the concentration of dissolved solids, primarily sugars, whereas titratable acidity (TA) represents the total concentration of organic acids and is commonly expressed as malic acid equivalents.

The ratio of soluble solids to titratable acidity (SSC/TA) is widely used as an indicator of flavour balance and maturity in fruits [22,23]. Titratable acidity varied more than twofold among cultivars, ranging from 2.38% in ‘Likernaja’ and 2.40% in ‘Burka’ to 5.36% in ‘Moravica’. The cultivars ‘Burka’ and ‘Likernaja’ exhibited significantly lower acidity than all other genotypes, whereas ‘Moravica’ had the highest acidity. Intermediate acidity levels were observed in most sweet rowanberry cultivars, including ‘Krasnaja’, ‘Kubovaja’, ‘Angri’, ‘Rossica’, and the wild rowanberry. The SSC/TA ratio varied significantly among cultivars, ranging from 3.33 in ‘Moravica’ to 6.68 in ‘Burka’. ‘Burka’ and ‘Oranzevaja’ exhibited the highest SSC/TA ratios, indicating the most favourable balance between sweetness and acidity, whereas ‘Moravica’ and ‘Rosina’ showed the lowest ratios and therefore a comparatively more acidic flavour profile. Intermediate ratios were observed in most cultivars, including ‘Krasnaja’, ‘Granatnaja’, ‘Rossica’, ‘Kubovaja’, and the wild rowanberry.

#### 2.1.4. Sugar and Organic Acid Composition in Berries and Processed Fractions

The concentrations of major soluble sugars (glucose and fructose), the sugar alcohol sorbitol, and major organic acids (malic, citric, shikimic, oxalic, and fumaric acids) were determined in three rowanberry fractions (juice, pomace, and whole fruit) across 17 cultivars.

**Figure 3:**
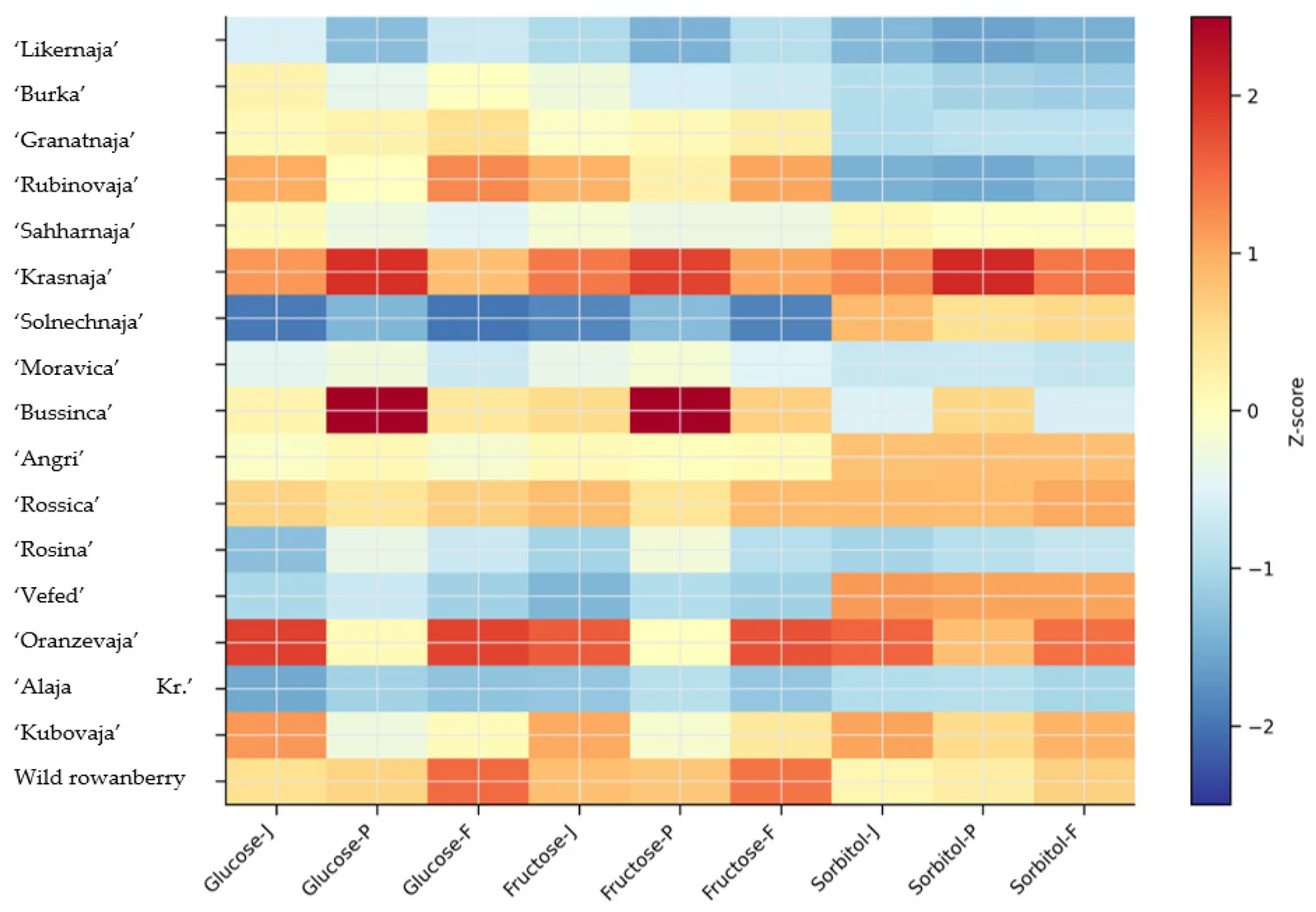
Sugar profiles of rowanberry cultivars across juice (J), pomace (P), and fruit (F) fractions. Colours represent standardized values (Z-scores), with red indicating relatively high values, blue indicating relatively low values, and white/yellow indicating intermediate values.

Across all sugars, the pomace fraction exhibited the highest concentrations, despite accounting for only approximately 10–35% of the fruit mass [30]. Intermediate concentrations were observed in the juice fraction, whereas the lowest concentrations were detected in the whole-fruit fraction (Figure 3). This distribution indicates that processing and fractionation significantly influence the concentration and partitioning of soluble carbohydrates. The higher sugar concentrations in pomace compared to juice and whole fruit can be explained by incomplete extraction and mass balance effects [31].

During juice processing, a fraction of sugars remains entrapped within the pomace matrix, as industrial processes aim but do not fully succeed in maximising the recovery of soluble solids. Pomace therefore remains rich in fermentable sugars (glucose and fructose), and additional pressing can recover significant amounts of juice, confirming that sugar-containing liquid remains within the residue [32]. In addition to incomplete extraction and mass-balance effects, the retention of soluble compounds within the structural matrix of the pomace may also contribute to the elevated concentrations observed. Fruit pomace is composed largely of plant cell-wall materials, including cellulose, hemicellulose, pectin, and lignin, which form a compact fibrous structure capable of retaining soluble compounds within residual fruit tissues [32]. Consequently, the enrichment observed in pomace is likely the result of both processing-related concentration effects and matrix-associated retention mechanisms.

Among the studied cultivars, the concentrations of sorbitol, glucose, and fructose showed a consistent pattern across all fractions, with ‘Krasnaja’, ‘Oranzevaja’, ‘Rossica’, ‘Kubovaja’ and ‘Vefed’ displaying the highest levels, while ‘Likernaja’, ‘Burka’, and ‘Granatnaja’ consistently showed the lowest values (Figure 3). Similar trends were reported by [25], suggesting that the consistent ranking across different fruit fractions indicates that sugars accumulate in a largely genotype-dependent manner.

The differences among cultivars were most pronounced in the pomace fraction, where fructose levels were the highest in ‘Bussinca’ and ‘Krasnaja’, 77.2 mg/g DW and 68.5 mg/g DW, respectively, and the lowest in ‘Likernaja’ and ‘Solnechnaja’, 34.4 mg/g DW and 35.3 mg/g DW. Similar trends were observed for glucose, with the highest concentrations recorded in ‘Bussinka’ and ‘Krasnaja’ (78.1 and 72.0 mg/g DW, respectively) and the lowest in ‘Solnechnaja’ and ‘Likernaja’ (39.2 and 40.0 mg/g DW, respectively) (Table S1 in Supplementary). The consistent patterns observed across fractions indicate that carbohydrate accumulation in rowanberry is strongly controlled by genetic factors. These findings highlight the importance of cultivar selection for optimising sugar composition in rowanberry processing and functional product development.

Sorbitol was the predominant soluble carbohydrate in all fractions and exhibited pronounced cultivar-dependent variation. Interestingly, the hybrid cultivars ‘Likernaja’, ‘Burka’, and ‘Granatnaja’ contained relatively low levels of soluble sugars but accumulated higher concentrations of shikimic acid. In contrast, cultivars with high sorbitol contents generally exhibited lower shikimic acid levels. As sorbitol metabolism and the shikimate pathway both rely on carbon originating from primary metabolism, these differences may reflect cultivar-specific patterns of carbon partitioning. In Rosaceae species, sorbitol serves as a major translocated and storage carbohydrate. In contrast, the shikimate pathway directs carbon flux towards aromatic amino acids and their derived secondary metabolites. The inverse relationship observed between sorbitol and shikimic acid concentrations therefore suggests that cultivars may differ in the relative allocation of carbon between carbohydrate accumulation and the synthesis of shikimate-pathway intermediates [33]. The co-occurrence of elevated shikimic acid and previously reported high phenolic levels in these cultivars [26] is consistent with a greater allocation of carbon towards shikimate-derived metabolites.

Although metabolic fluxes were not measured directly, this observation suggests increased allocation of photosynthetically derived carbon towards secondary-metabolite biosynthesis rather than carbohydrate storage. Further studies using metabolic flux analysis and transcriptomic approaches would be required to confirm this hypothesis.

**Figure 4:**
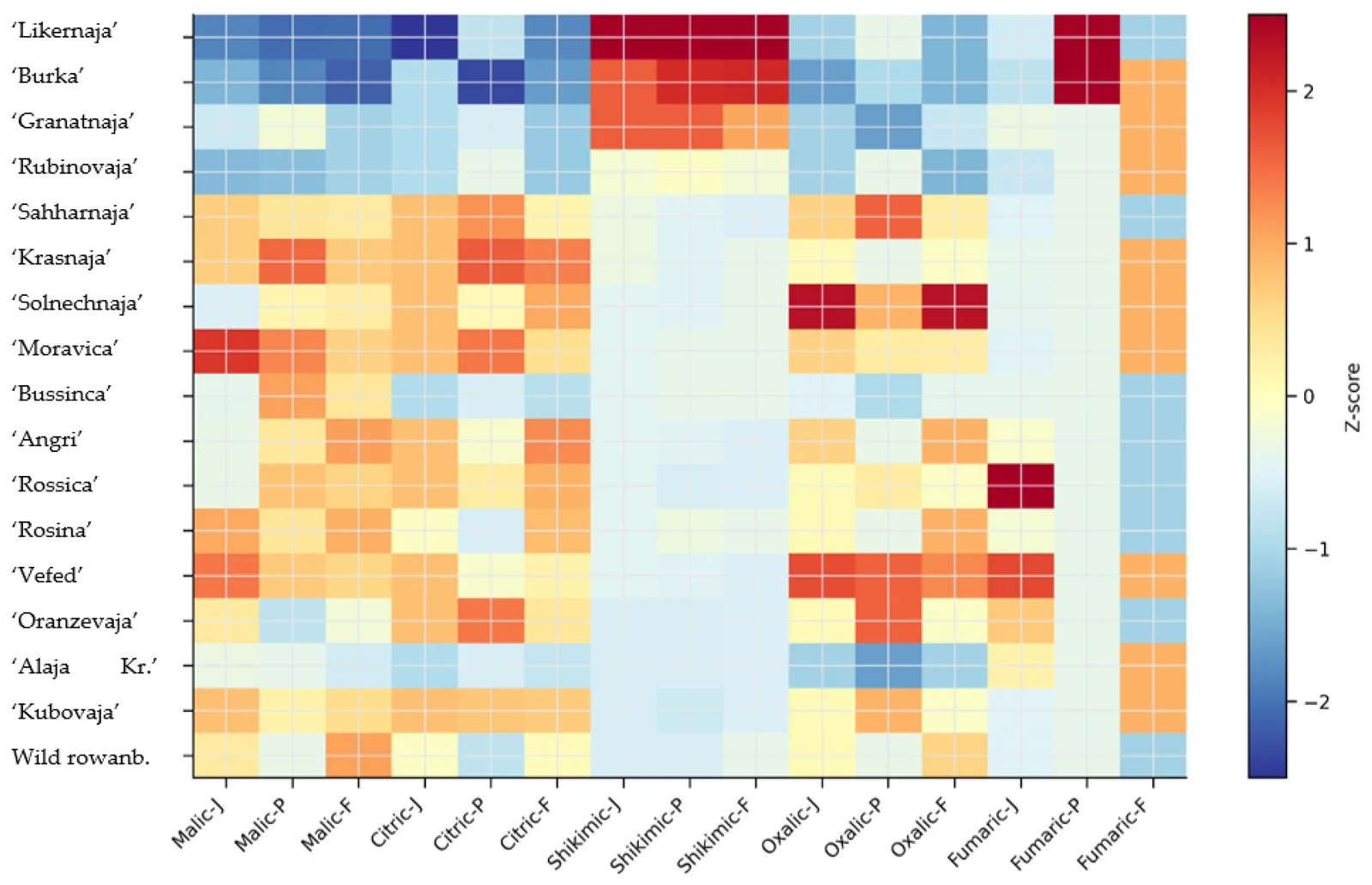
Organic acid profiles of rowanberry cultivars across juice (J), pomace (P), and fruit (F) fractions. Colours represent standardized values (Z-scores), with red indicating relatively high values, blue indicating relatively low values, and white/yellow indicating intermediate values.

Indeed, a distribution pattern similar to that observed for sugars was evident for organic acids (Figure 4), with higher concentrations generally detected in pomace than in juice or whole fruit. This effect can be attributed to mass-balance and matrix-retention phenomena during juice extraction, where incomplete release of soluble compounds and reduced water content in the pomace fraction resulted in elevated concentrations on a dry-weight basis. The parallel enrichment of both sugars and organic acids in pomace highlights the strong influence of processing on metabolite partitioning and demonstrates the importance of considering both genotype and processing fraction when evaluating rowanberry fruit composition.

Across organic acids, malic acid was the dominant component in all fractions, with the highest concentrations observed in the pomace and whole-fruit fractions of ‘Krasnaja’ (up to 70.9 mg/g DW), whereas juice contained comparatively lower levels (Figure 4). In contrast, ‘Likernaja’ and ‘Burka’ consistently exhibited the lowest malic acid concentrations.

Citric acid showed marked variability and was particularly abundant in the whole-fruit fraction, reaching 1.21 mg/g DW in ‘Krasnaja’, suggesting a contribution to cultivar-specific acidity.

Oxalic acid exhibited moderate variation, with higher levels (0.10 mg/g DW) observed in ‘Sahharnaja’, ‘Oranzevaja’, and ‘Vefed’, particularly in the pomace and whole fruit fractions, while ‘Burka’, ‘Likernaja’, and ‘Alaja Krupnaja’ had the lowest levels. Fumaric acid showed the lowest concentrations and the least variability among all analysed acids, confirming its minor role in differentiating cultivars.

When sugar and organic acid profiles are considered together, clear genotype-dependent differences in metabolite allocation become apparent. Cultivars such as ‘Krasnaja’, ‘Oranzevaja’, ‘Rossica’, ‘Kubovaja’, and ‘Vefed’ preferentially accumulated soluble carbohydrates, particularly sorbitol, whereas ‘Likernaja’, ‘Burka’, and ‘Granatnaja’ exhibited lower sugar concentrations but higher shikimic acid levels. The observed variation in organic-acid pools further suggests differences in the regulation of primary carbon metabolism among cultivars. These compositional differences were reflected in SSC/TA ratios, which integrate the effects of sugar accumulation and acidity and therefore influence flavour balance and processing suitability. Furthermore, cultivar-dependent colour characteristics may be associated with differences in the accumulation of shikimate-derived phenolic pigments, as indicated by the relationship between elevated shikimic acid concentrations and previously reported high phenolic contents [26]. Collectively, these findings indicate coordinated partitioning of carbon between soluble sugars, organic acids, and secondary metabolites, ultimately influencing fruit quality, technological properties, and the suitability of rowanberry cultivars for juice production, fermentation, and functional food applications.

## 3. Discussion

The present study revealed substantial variation among rowanberry genotypes in fruit colour, sugar composition, organic acid content, sorbitol concentration, and shikimate pathway metabolites. These differences demonstrate the strong influence of genotype on fruit quality traits and highlight the considerable biochemical diversity present within cultivated and wild rowanberry germplasm. The observed variation in sugars and organic acids is particularly important because these compounds largely determine fruit taste and consumer perception [23,34]. Cultivars with higher sugar contents and lower acidity are generally more suitable for fresh consumption. In contrast, genotypes rich in organic acids may be valuable for juice production, blending, or the manufacture of products requiring a distinctive flavour profile. The considerable differences observed among genotypes indicate that cultivar selection may strongly influence the sensory properties and technological performance of rowanberry-based products. Consequently, sugar and organic acid composition should be considered important selection criteria in breeding programmes and in the development of products targeted to specific consumer preferences.

Sorbitol represented a major component of carbohydrate metabolism in rowanberry fruits. As the principal translocated carbohydrate in many Rosaceae species, sorbitol plays an important role in carbon transport and storage and may influence fruit development and quality [35]. The substantial differences observed among genotypes indicate possible genetic variation in carbon allocation and metabolic regulation.

Variation in shikimic acid content among the studied genotypes may reflect differences in the activity of metabolic pathways involved in the biosynthesis of aromatic compounds and other biologically important metabolites. As the shikimate pathway serves as a central metabolic route linking primary and secondary metabolism, the observed variation provides valuable insights into the biochemical characteristics of rowanberry fruits [33].

The results also demonstrated that considerable amounts of sugars and organic acids remained in the pomace fraction after juice extraction. This finding indicates that rowanberry pomace retains valuable compositional characteristics in addition to the dietary fibre, pectin, minerals, and phenolic compounds reported previously [26,27]. The presence of these compounds may contribute to flavour, sweetness, and acidity, supporting the utilisation of pomace as a value-added ingredient in functional foods, fruit-based products, and nutraceutical formulations. The recovery and utilisation of pomace could therefore enhance resource efficiency and contribute to the sustainable valorisation of rowanberry processing by-products.

Although rowanberries are widely recognised as rich sources of phenolic compounds and other bioactive constituents, the present study focused specifically on sugars and organic acids because these compounds are key determinants of fruit flavour, sweetness, acidity, and overall processing suitability. In addition, sugars and organic acids are directly associated with consumer acceptance and technological properties of raw materials intended for juice, jam, and other fruit-product manufacture. The phenolic composition, antioxidant capacity, and related bioactive characteristics of many of the investigated cultivars have previously been described [26,27]. Consequently, the present study complements earlier findings by providing detailed information on primary metabolites that are important for fruit quality and processing performance.

Pearson correlation analysis revealed a very strong positive correlation between SSC and sorbitol concentration (r = 0.991, *p* < 0.001), indicating that differences in SSC among rowanberry genotypes were largely associated with sorbitol accumulation. Glucose and fructose contents were also strongly correlated (r = 0.968, *p* < 0.001), suggesting coordinated accumulation of these sugars among genotypes. Furthermore, SSC showed significant positive correlations with titratable acidity (r = 0.649, *p* = 0.005) and fruit colour parameters, including L (r = 0.671, *p* = 0.003), b (r = 0.752, *p* < 0.001), and h° (r = 0.790, *p* < 0.001). These relationships indicate an association between sugar accumulation, acidity, and fruit colour characteristics in rowanberry fruits (Supplementary Table S3).

Overall, the results demonstrate that rowanberry genetic resources contain considerable diversity that could be exploited in breeding programmes aimed at developing cultivars with improved sensory quality, nutritional value, and suitability for different processing applications.

The present study has several strengths and limitations that should be considered when interpreting the results. A major strength is the comprehensive evaluation of 17 rowanberry genotypes, including sweet cultivars, interspecific hybrids, and a wild genotype, grown under the same environmental conditions. This experimental design enabled direct comparison of genotypic differences in fruit colour, sugars, organic acids, and related quality traits while minimising environmental variation. In addition, the simultaneous assessment of whole fruits, juice, and pomace fractions provided valuable information relevant to fruit processing and by-product valorisation. However, as all genotypes were evaluated at a single location during one growing season, the potential influence of year-to-year climatic variation and genotype × environment interactions could not be fully assessed. Therefore, the observed patterns should be interpreted within the context of the growing conditions of the study.

Future studies should investigate the stability of sugar, organic acid, sorbitol, and shikimate pathway metabolite accumulation across multiple years and growing environments. A better understanding of genotype × environment interactions would provide valuable information for cultivar selection and commercial production. Monitoring the dynamics of sugars, organic acids, sorbitol, and shikimic acid throughout fruit development would provide further insight into the metabolic changes occurring during ripening and their contribution to final fruit quality. Furthermore, integrating metabolomic, transcriptomic, and sensory analyses could improve understanding of the mechanisms underlying cultivar-specific differences in fruit quality. Particular attention should be given to the role of shikimic acid and related metabolites, including phenylalanine, tyrosine, and tryptophan, in the biosynthesis of aroma compounds and other quality-associated constituents [36]. Such studies would contribute to the development of rowanberry cultivars with enhanced commercial and nutritional value.

## 4. Materials and Methods

### 4.1. Plant Material and Fraction Separation

Seventeen rowanberry (*Sorbus* spp.) genotypes, comprising sweet cultivars, interspecific hybrids, and a wild rowanberry (*S. aucuparia*), were included in the study. Fully ripe fruits were harvested under comparable growing conditions from the Polli Experimental Station (Estonia). After harvesting, the fruits were immediately frozen and stored at −20 °C until further analysis.

Frozen fruits were thawed prior to processing. Juice was extracted using a low-speed juicer (SJF01CREU; Smeg S.p.A., Guastalla, Italy). The juice yield ranged from 70 to 75% of the fresh fruit weight. Three fractions were obtained for subsequent analyses: whole fruit, juice, and pomace.

The rowanberries, juice, and pomace were frozen at −40 ± 2 °C and freeze-dried using an Advantage Plus Benchtop Freeze Dryer (SP Industries, Warminster, PA, USA) at 30 μbar for 72 h. Pomace samples were subsequently ground using a Retsch Mixer Mill MM 400 (Retsch GmbH, Haan, Germany) equipped with ZrO_2_ grinding balls for 1.5 min at 30 Hz. Freeze-dried samples were stored in airtight tubes at −25 °C until analysis.

### 4.2. Physical and Basic Chemical Analysis

Fruit mass was determined using 30 fruits collected from different clusters and trees of each genotype. The fruits were weighed, and the average fruit mass (g) was calculated by dividing the total sample weight by the number of fruits. Measurements were performed in triplicate.

Fruit colour was measured using a Minolta CR-400 colorimeter (Konica Minolta, Langenhagen, Germany). Colour parameters were recorded in the CIELAB colour space, where L* represents lightness, a* the red–green axis, and b* the yellow–blue axis. Chroma (C*) and hue angle (h°) were calculated to describe colour saturation and hue, respectively.

Total soluble solids (SCC) were determined using a digital refractometer (Atago Co., Ltd., Tokyo, Japan) and expressed as °Brix. Titratable acidity (TA) was determined by titrating 5 mL of juice diluted with distilled water to a final volume of 100 mL using 0.1 M NaOH to an endpoint pH of 8.1. Results were expressed as percentage malic acid equivalents.

### 4.3. Sugar and Organic Acid Analysis

Soluble sugars and organic acids were analysed using a Shimadzu Nexera X2 chromatographic system (Shimadzu, Kyoto, Japan) equipped with LC-30AD pumps, a SIL-30AC autosampler, and a CTO-20AC column oven. All samples were filtered through 0.22 μm membrane filters prior to analysis.

Soluble sugars (glucose, fructose, and sorbitol) were separated on a Luna Omega column (3 μm, 100 Å, 250 × 4.6 mm; Phenomenex, Torrance, CA, USA) maintained at 40 °C. The mobile phase consisted of acetonitrile and water (77:23, *v*/*v*) supplied isocratically at a flow rate of 1.0 mL min^−1^. The autosampler temperature was set to 15 °C and the injection volume was 4 μL. Sugars were detected using an RID-20A refractive index detector operated at 40 °C. Identification was based on comparison of retention times with authentic standards, and quantification was performed using external calibration curves.

Organic acids (malic, citric, fumaric, shikimic, and oxalic acid) were determined using an Ultra-High-Performance Liquid Chromatography (UHPLC) system coupled with a photodiode array detector and electrospray ionisation mass spectrometric detection (PDA–ESI-MS). Chromatographic separation was carried out on an XSelect HSS T3 C18 column (150 × 4.6 mm, 2.2 μm; Waters, Milford, MA, USA) maintained at 30 °C. The mobile phase consisted of 0.1% formic acid in water (A) and acetonitrile (B) delivered at a flow rate of 0.40 mL min^−1^. Gradient elution was performed as follows: 0–3 min, 2% B; 3–7 min, 80% B; 7–12 min, 80% B; 12–13 min, 2% B; followed by re-equilibration until 18 min. Samples were maintained at 4 °C prior to injection. Detection was performed over the wavelength range of 200–800 nm. The ESI source was operated at an interface temperature of 350 °C, desolvation line temperature of 300 °C, heat block temperature of 400 °C, nebulising gas flow of 3.0 L min^−1^, and drying gas flow of 14.0 L min^−1^. Organic acids were identified by comparison of retention times and mass spectral characteristics with those of reference standards and quantified using external calibration curves. Results were expressed as mg g^−1^ dry weight (DW).

### 4.4. Statistical Analysis

All analyses were performed in triplicate, and the results are presented as mean ± standard deviation (SD). One-way analysis of variance (ANOVA) was used to evaluate the effect of genotype on the studied physical and chemical parameters. When significant differences were detected, mean values were compared using Tukey’s honestly significant difference (HSD) test at a significance level of *p* < 0.05.

Hierarchical cluster analysis (HCA) based on CIELAB colour parameters (L*, a*, b*, C*, and h°) was performed using Ward’s method to classify genotypes according to fruit colour characteristics. Heatmaps were generated using Microsoft Excel 365 (Microsoft Corp., Redmond, WA, USA).

## 5. Conclusions

The results revealed substantial genotype-dependent variation in sugar composition, organic acid content, SSC/TA ratio, and fruit colour characteristics among rowanberry cultivars. Several cultivars, particularly ‘Krasnaja’, ‘Oranzevaja’, ‘Rossica’, ‘Kubovaja’, and ‘Vefed’, were characterised by high concentrations of soluble carbohydrates, whereas ‘Likernaja’, ‘Burka’, and ‘Granatnaja’ exhibited lower sugar contents and higher shikimic acid concentrations. These differences influenced fruit quality attributes and demonstrated considerable biochemical diversity within rowanberry germplasm. The findings provide valuable information for cultivar selection, food-processing applications, and the valorisation of rowanberry processing by-products.

## Figures and Tables

**Table 1:** Soluble solids content (SSC), titratable acidity (TA), and the SSC/TA ratio of rowanberry cultivars and wild rowanberry.

Cultivar	SSC (°Brix)	TA (% Malic Acid Equivalents)	SSC/TA Ratio
‘Burka’	16.0± 0.06 ^j^	2.40 ± 0.02 ^l^	6.68 ± 0.07 ^a^
‘Likernaja’	14.5± 0.06 ^l^	2.38 ± 0.03 ^l^	6.11 ± 0.07 ^b^
‘Granatnaja’	17.6± 0.12 ^i^	3.26 ± 0.02 ^j^	5.42 ± 0.03 ^d^
‘Rubinovaja’	16.4 ± 0.1 ^j^	3.07 ± 0.03 ^k^	5.33 ± 0.06 ^de^
‘Alaja Krupnaja’	15.5 ± 0.1 ^k^	3.78 ± 0.01 ^i^	4.11 ± 0.01 ^j^
‘Moravica’	17.9 ± 0.1 ^i^	5.36 ± 0.02 ^a^	3.33 ± 0.02 ^l^
‘Krasnaja’	27.4 ± 0.1 ^b^	4.81 ± 0.05 ^ef^	5.69 ± 0.04 ^c^
‘Kubovaja’	25.5 ± 0.2 ^d^	4.92 ± 0.03 ^de^	5.18 ± 0.06 ^f^
‘Oranzevaja’	27.9 ± 0.0 ^a^	4.19 ± 0.05 ^h^	6.66 ± 0.03 ^a^
‘Sahharnaja’	20.5 ± 0.1 ^g^	4.73 ± 0.03 ^f^	4.34 ± 0.04 ^i^
‘Vefed’	25.4 ± 0.1 ^de^	5.19 ± 0.05 ^b^	4.89 ± 0.03 ^g^
‘Rossica’	26.5 ± 0.0 ^c^	5.09 ± 0.04 ^c^	5.21 ± 0.05 ^ef^
‘Solnechnaja’	23.2 ± 0.1 ^f^	5.06 ± 0.05 ^c^	4.59 ± 0.04 ^h^
‘Angri’	25.3 ± 0.0 ^de^	4.82 ± 0.04 ^ef^	5.25 ± 0.04 ^ef^
‘Bussinka’	19.5 ± 0.1 ^h^	4.63 ± 0.06 ^g^	4.22 ± 0.04 ^ij^
‘Rosina’	18.0 ± 0.0 ^i^	5.03 ± 0.02 ^cd^	3.58 ± 0.01 ^k^
Wild rowanberry	25.2 ± 0.1 ^e^	4.84 ± 0.03 ^ef^	5.21 ± 0.03 ^ef^

Values are presented as mean ± SD (*n* = 3). The SSC/TA ratio was calculated from individual replicate values of soluble solids content (SSC, °Brix) and titratable acidity (TA, % malic acid equivalents). Different letters indicate significant differences according to Tukey’s HSD test.

## Data Availability

The data presented in this study are available in the article and Supplementary Materials. Additional data supporting the findings of this study are available from the corresponding author upon reasonable request.

## Supplementary Materials

The following supporting information can be downloaded at: https://www.mdpi.com/article/10.3390/molecules31162796/s1, Table S1: The content of individual sugars and (b) organic acids in fruit, juice and pomace of 17 rowanberry cultivars (mg/g DW); Table S2: The content of organic acids in fruit, juice and pomace of 17 rowanberry cultivars (mg/g DW); Table S3. Significant Pearson correlations among SSC, titratable acidity, sugar composition, and fruit colour parameters of rowanberry genotypes.
