# Supplementary material for: Compositional Variability of Rowanberry Genotypes and Sugar–Organic Acid Distribution Between Juice and Pomace
Source: Molecules. 2026 Aug 11;31(16):2796. doi: 10.3390/molecules31162796 (PMC13515838; doi:10.3390/molecules31162796)
Supplement: Supplementary file 1 [file molecules-31-02796-s001.zip › molecules-4452630-supplementary.pdf]

Supplementary Tables

Supplementary Table S1. The content of individual sugars and (b) organic acids in fruit, juice and pomace of 17 rowanberry cultivars (mg/g DW).

	**Glucose**			**Fructose**			**Sorbitol**		
**Cultivar**	**Juice**	**Pomace**	**Fruit**	**Juice**	**Pomace**	**Fruit**	**Juice**	**Pomace**	**Fruit**
‘Burka‘	36.0 ± 0.8bc	48.9± 0.7^{fg}	28.8± 1.0^{cd}	33.5± 0.8^{c}	43.0± 1.0^{c}	29.1± 1.6^{f}	53.8± 1.1hi	66.5± 0.3^{i}	47.9± 2.3^{h}
‘Likernaja‘	32.6± 0.8c	40.0± 2.9^{i}	25.8± 1.3^{ef}	30.0± 0.6^{d}	34.4± 1.9^{d}	28.0± 0.9^{f}	40.7± 1.4^{k}	46.2± 0.3^{j}	39.2± 0.7^{i}
‘Granatnaja‘	35.6± 1.2bc	54.5± 1.7^{de}	31.0± 1.3^{bc}	34.4± 0.6^{c}	50.2± 0.9^{c}	33.9± 0.9^{cd}	52.5± 2.5^{i}	75.2± 0.4^{h}	56.1± 1.8^{g}
‘Rubinovaja‘	39.7± 1.2ab	52.8± 1.8^{de}	34.5± 1.9^{ab}	39.2± 1.9^{b}	51.5± 1.4^{b}	38.0± 0.9^{ab}	38.4± 1.2^{k}	48.6± 0.2^{j}	42.2± 1.1^{i}
‘Alaja Krupnaja‘	28.2± 0.9d	42.4± 1.0^{h}	23.3± 2.5^{g}	28.8± 1.4^{d}	40.0± 0.6^{d}	26.4± 1.0^{g}	53.9± 2.2^{hi}	73.5± 0.1^{h}	50.9± 2.7^{h}
‘Moravica‘	33.2± 1.3c	50.3± 2.2^{ef}	25.8± 0.9^{ef}	33.0± 0.6^{c}	47.4± 1.9^{c}	30.0± 0.8^{ef}	59.8± 1.2^{h}	80.7± 3.0^{g}	57.3± 4.1^{fg}
‘Krasnaja‘	40.5± 1.8ab	72.0± 1.2^{b}	32.4± 2.4^{abc}	41.5± 0.6^{a}	68.5± 1.0^{a}	38.0± 0.6^{ab}	118.0± 1.8^{b}	189.0± 0.2^{a}	116.8± 3.3^{a}
‘Kubovaja‘	40.5± 0.7ab	50.1± 1.8^{ef}	29.0± 2.6^{cd}	39.7± 1.0^{a}	47.7± 1.0^{a}	34.5± 0.8^{cd}	111.9± 3.2^{cd}	128.9± 0.6^{d}	103.7± 1.4^{bc}
‘Oranzevaja‘	43.8± 1.9a	53.4± 2.3^{de}	36.9± 4.1^{a}	42.7± 1.3^{a}	49.3± 0.9^{a}	41.4± 2.4^{a}	126.2± 1.5^{a}	139.9± 0.4^{c}	118.1± 3.8^{a}
‘Sahharnaja‘	35.5± 1.3bc	50.0± 1.3^{ef}	26.8± 2.7^{de}	33.9± 1.4^{c}	46.1± 0.2^{c}	31.0± 0.9 ^{e}	83.9± 1.8^{f}	106.7± 0.2^{f}	76.9± 0.6^{e}
‘Vefed‘	30.6± 0.9d	46.0± 1.7^{gh}	24.0± 2.9^{fg}	28.0± 0.9^{d}	39.6± 1.2^{d}	27.0± 0.8^{fg}	113.9± 2.8^{c}	150.0± 1.2^{b}	107.2± 4.2^{b}
‘Rossica‘	38.0± 2.2b	56.6± 0.8^{cd}	31.7 ± 2.4^{abc}	38.8± 1.3^{b}	53.5± 1.1^{b}	37.0± 0.5^{b}	106.2± 1.1^{d}	141.8± 1.9^{c}	106.2± 1.5^{b}
‘Solnechnaja‘	26.2± 1.6e	39.2± 1.9^{i}	20.1± 2.0^{h}	25.8± 1.1^{e}	35.3± 0.8^{e}	23.0± 0.2^{h}	106.2± 0.8^{e}	127.1± 1.8^{d}	93.7± 1.5^{cd}
‘Angri‘	34.9± 1.1c	53.7± 1.5^{de}	28.2± 1.5^{d}	35.1± 0.3^{c}	49.6± 1.2^{c}	33.0± 1.1^{de}	103.5± 1.1^{de}	140.2± 1.9^{c}	100.4± 2.5^{c}
‘Bussinca‘	35.9± 1.6bc	78.1± 1.9^{a}	30.4± 1.7^{bc}	37.3± 0.8^{b}	77.2± 1.2^{b}	36.0± 0.1^{bc}	65.5± 2.2^{g}	131.1± 6.9^{d}	62.7± 0.6^{f}
‘Rosina‘	29.3± 0.9d	49.3± 2.3^{fg}	25.8± 0.2^{ef}	29.6± 0.5^{d}	46.8± 1.0^{d}	28.0± 0.5^{f}	49.6± 1.7^{j}	74.2± 1.2^{h}	58.1± 1.3^{fg}
Wild rowanberry	37.4± 1.6b	58.6± 3.2^{c}	35.5± 2.2^{ab}	38.7± 0.9^{b}	57.1± 1.1^{b}	40.0± 0.5^{a}	84.5± 2.5^{f}	119.3± 4.1^{e}	96.0± 1.5^{cd}

Supplementary Table S2.The content of organic acids in fruit, juice and pomace of 17 rowanberry cultivars (mg/g DW).

	**Malic acid**			**Citric acid**			**Shikimic acid**			**Oxalic acid**			**Fumaric acid**		
**Cultivar**	**Juice**	**Pomace**	**Fruit**	**Juice**	**Pomace**	**Fruit**	**Juice**	**Pomace**	**Fruit**	**Juice**	**Pomace**	**Fruit**	**Juice**	**Pomace**	**Fruit**
**‘Likernaja‘**	5.8^{f}	35.6^{h}	33.2^{i}	0.001± 0.001^{h}	0.12± 0.001^{g}	0.38± 0.001^{g}	0.03± 0.002^{a}	0.041± 0.003^{a}	0.028± 0.003^{a}	0.002± 0.000^{g}	0.07± 0.001^{cd}	0.003± 0.000^{g}	0.000	0.002± 0.000^{a}	0.001± 0.000^{b}
**‘Burka‘**	7.2e^{f}	37.8^{h}	32.2^{i}	0.02± 0.000^{g}	0.05± 0.001^{h}	0.43± 0.001^{g}	0.02± 0.002^{b}	0.035± 0.000^{b}	0.023± 0.003^{b}	0.001± 0.000^{h}	0.06± 0.001^{e}	0.003± 0.001^{g}	0.000	0.002± 0.000^{a}	0.002± 0.000^{a}
**‘Granatnaja‘**	9.5^{de}	53.8^{ef}	42.9^{gh}	0.02± 0.006^{g}	0.13± 0.008^{fg}	0.55± 0.001^{fg}	0.02± 0.003^{c}	0.031± 0.002^{c}	0.017± 0.001^{c}	0.002± 0.000 ^{g}	0.05± 0.01^{fg}	0.005± 0.001^{fg}	0.000	0.001± 0.000^{b}	0.002± 0.000^{a}
**‘Rubinovaja‘**	7.4^{ef}	43.2^{gh}	43.0^{gh}	0.02± 0.001^{g}	0.14± 0.007^{ef}	0.55± 0.002^{fg}	0.007± 0.000^{d}	0.015± 0.000^{d}	0.010± 0.003^{d}	0.002± 0.000^{g}	0.07± 0.001^{cd}	0.003± 0.000^{g}	0.000	0.001± 0.000^{b}	0.002± 0.000^{a}
**‘Sahharnaja‘**	13.7^{b}	59.7^{cd}	56.8^{de}	0.04± 0.000^{bc}	0.21± 0.006^{ab}	0.90± 0.001^{de}	0.006± 0.00^{d}	0.011± 0.001^{fg}	0.008± 0.0001^{ef}	0.005± 0.000^{cd}	0.10± 0.02^{a}	0.008± 0.000^{cd}	0.000	0.001± 0.000^{b}	0.001± 0.000^{b}
**‘Krasnaja‘**	13.7^{b}	70.9^{a}	60.7^{bc}	0.04± 0.002^{a}	0.23± 0.007^{a}	1.21± 0.001^{a}	0.006± 0.0004^{e}	0.011± 0.002^{fg}	0.009± 0.002^{de}	0.004± 0.000 ^{de}	0.07± 0.001^{cd}	0.007± 0.000^{de}	0.000	0.001± 0.000^{b}	0.002± 0.000^{a}
**‘Solnechnaja‘**	9.9d^{e}	57.2^{de}	56.4^{de}	0.04± 0.001^{a}	0.16± 0.006^{cd}	1.13± 0.001^{ab}	0.005± 0.000^{ef}	0.011± 0.002^{fg}	0.009± 0.003^{de}	0.008± 0.000^{a}	0.09± 0.01^{b}	0.014± 0.000^{a}	0.000	0.001± 0.000^{b}	0.002± 0.000^{a}
**‘Moravica‘**	17.7^{a}	68.5^{ab}	60.0^{bc}	0.04± 0.002^{cd}	0.22± 0.005^{ab}	0.99± 0.001^{cd}	0.005± 0.000^{ef}	0.012± 0.002^{ef}	0.009± 0.001^{de}	0.005± 0.000^{cd}	0.08± 0.01^{bc}	0.008± 0.000^{cd}	0.000	0.001± 0.000^{b}	0.002± 0.000^{a}
**‘Bussinca‘**	10.3^{cd}	66.4^{bc}	57.5^{de}	0.02± 0.000^{fg}	0.13± 0.005^{fg}	0.63± 0.002^{f}	0.005± 0.000^{f}	0.012± 0.000^{ef}	0.009± 0.001^{de}	0.003± 0.000^{f}	0.06± 0.01^{e}	0.006± 0.001^{ef}	0.000	0.001± 0.000^{b}	0.001± 0.000^{b}
**‘Angri‘**	10.5^{cd}	59.3^{cd}	64.7^{a}	0.04± 0.001^{ab}	0.15± 0.005^{e}	1.19± 0.003^{a}	0.005± 0.000^{fg}	0.011± 0.001^{fg}	0.008± 0.002^{ef}	0.005± 0.000^{cd}	0.07± 0.001^{cd}	0.010± 0.001^{bc}	0.000	0.001± 0.000^{b}	0.001± 0.000^{b}
**‘Rossica‘**	10.5^{cd}	63.4^{bc}	59.7^{bc}	0.04± 0.001^{bc}	0.17± 0.004^{cd}	1.11± 0.002^{ab}	0.005± 0.000^{fg}	0.010± 0.000^{gh}	0.008± 0.000^{ef}	0.004± 0.000^{de}	0.08± 0.001^{bc}	0.007± 0.001^{de}	0.000	0.001± 0.000^{b}	0.001± 0.000^{b}
**‘Rosina‘**	14.8^{ab}	59.8^{cd}	63.2^{ab}	0.03± 0.000^{e}	0.13± 0.02^{fg}	1.08± 0.002^{ab}	0.005± 0.001^{fg}	0.013± 0.001^{e}	0.009± 0.003^{de}	0.004± 0.001^{de}	0.07± 0.01^{cd}	0.010± 0.001^{bc}	0.000	0.001± 0.000^{b}	0.001± 0.000^{b}
**‘Vefed‘**	16.0^{ab}	62.9^{bc}	59.3^{cd}	0.04± 0.004^{bc}	0.15± 0.006^{de}	0.91± 0.09^{de}	0.005± 0.000^{g}	0.011± 0.001^{fg}	0.008± 0.001g	0.007± 0.000^{b}	0.10± 0.02^{a}	0.011± 0.001^{b}	0.000	0.001± 0.000^{b}	0.002± 0.000^{a}
**‘Oranzevaja‘**	1267^{bc}	48.1^{fg}	51.3^{ef}	0.04± 0.005^{d}	0.22± 0.005^{ab}	96.9± 0.05^{cd}	0.004± 0.001^{g}	0.010± 0.001^{gh}	0.008± 0.000^{ef}	0.004± 0.000^{de}	0.10± 0.01^{a}	0.007± 0.001^{de}	0.000	0.001± 0.000^{b}	0.001± 0.000^{b}
**‘Alaja Kr.‘**	10.6^{cd}	52.0^{ef}	47.5^{fg}	0.02± 0.001^{g}	0.13± 0.003^{fg}	0.66± 0.04f	0.004± 0.000^{gh}	0.010± 0.000^{gh}	0.008± 0.001^{ef}	0.002± 0.000^{g}	0.05± 0.01^{fg}	0.004± 0.000^{g}	0.000	0.001± 0.000^{b}	0.002± 0.000^{a}
**‘Kubovaja‘**	14.1^{b}	57.8^{de}	58.7^{cd}	0.04± 0.002^{cd}	0.19± 0.005^{bc}	1.04± 0.001^{bc}	0.004± 0.000^{gh}	0.009± 0.001^{h}	0.008± 0.001^{ef}	0.004± 0.000^{de}	0.09± 0.001^{b}	0.007± 0.001^{de}	0.000	0.001± 0.000^{b}	0.002± 0.001^{abc}
**Wild rowan-berry**	12.6^{bc}	52.4^{ef}	64.4^{a}	0.03± 0.001^{f}	0.12± 0.004^{g}	0.87± 0.002^{de}	0.004± 0.000^{h}	0.010± 0.001^{gh}	0.009± 0.001^{de}	0.004± 0.000^{de}	0.07± 0.001^{cd}	0.009± 0.001^{cd}	0.000	0.001± 0.000^{b}	0.001± 0.000^{c}

Supplementary Table S3. Significant Pearson correlations among SSC, titratable acidity, sugar composition, and fruit colour parameters of rowanberry genotypes.

Variables	R	p-value
SSC vs Sorbitol	0.991	<0.001
SSC vs h°	0.790	<0.001
SSC vs b*	0.752	<0.001
SSC vs L*	0.671	0.003
SSC vs TA	0.649	0.005
Glucose vs Fructose	0.968	<0.001
